# The Treatment of Uterine Flexions

**Published:** 1886-08

**Authors:** Virgil O. Hardon

**Affiliations:** Lecturer on Operative Gynæcology, Southern Medical College, Atlanta, Ga.


THE ATLANTA MEDICAL AND SURGICAL JOURNAL.

VOL. III. AUGUST, 1886. No. 6.

### Original Communications.

### THE TREATMENT OF UTERINE FLEXIONS.

BY VIRGIL O. HARDON, M. D., LECTURER ON OPERATIVE GYNÆCOLOGY, SOUTHERN MEDICAL COLLEGE, ATLANTA, GA.

In a paper read before the Atlanta Society of Medicine, October 20, 1885,* I gave a description of a mechanical device which was offered as a substitute for pessaries in the treatment of uterine displacements. It is proposed in this paper to elaborate in greater detail the principles upon which that method is based, and the manner of its application to the treatment of flexions, to which class of displacements it is especially applicable.

It is a matter of common observation that cases are now and then seen in which a marked flexion of the womb persists for a long time without producing any symptoms. It is extremely probable that such instances are much more common than is usually supposed, since it is only by accident that they come under the notice of the physician. In such cases the organ is found

* *Atlanta Medical and Surgical Journal*, December, 1885.

high up in the pelvis, almost beyond the reach of the examining finger and the flexion is usually forward. I have never seen this condition in any other than nulliparous women in whom the uterine ligaments and the vaginal walls have retained their virgin firmness and tone. In such cases there is no tenderness or enlargement of the organ, no interference with the functions of the bladder, and no pain, except perhaps a tendency to dysmenorrhœa. If flexion of the womb may persist for years without producing any symptoms, this fact would seem to demonstrate that it does not constitute a disease *per se*, and that of itself it is incapable of producing the symptoms usually attributed to it.

But, on the other hand, the majority of cases of uterine flexion which are brought to the notice of the physician present a striking contrast to the foregoing picture and are accompanied by a group of painful symptoms which demand active treatment for their relief. Pain in the back, difficulty of locomotion, sense of fullness in the pelvis, dragging sensations in the loins, vesical tenesmus, frequent or painful micturition, leucorrhœa, dysmenorrhœa, menorrhagia and metrorrhagia are the principal features of such cases. Vaginal examination shows the womb to be low down in the pelvis, tender to the touch, its walls thickened so that the whole organ is enlarged, though usually of normal depth, and its endo-cervical mucous membrane in a state of chronic inflammation. This condition is most commonly seen in women who have borne children, and in such cases there will, as a rule, be found associated with it some accident of parturition, such as laceration of the cervix, rupture of the perineum, or injury to the vesico-vaginal or recto-vaginal septum. In those less frequent cases in which this condition presents itself in a nulliparous woman, there usually exists a stenosis of the cervical canal, a uterine polypus or a fibroid tumor.

There are found, then, two groups of cases of uterine flexion differing essentially with each other as regards clinical history. The one presents a variety of morbid symptoms of such severity that the patient is a decided invalid and seeks medical aid. The other presents an entire absence of symptoms, and the condition is brought to the notice of the physician only by accident. What factor determines this marked difference? The study of this question cannot fail to throw light upon the proper treatment of uterine flexions.

The symptoms which are characteristic of the first group of cases, and which form a *tout ensemble* which every practitioner will at once recognize from his own clinical experience, are attributable to three causes:

1. Traction upon the uterine supports
2. Obstruction of the uterine circulation.
3. Traction upon the bladder.

In the first class are found pain in the back, dragging sensations in the loins, difficulty of locomotion; in the second, leucorrhœa, dysmenorrhœa, menorrhagia, metrorrhagia, sense of fullness in the pelvis; and in the third, vesical tenesmus and frequent and painful micturition. I shall endeavor to show that these three causative conditions have their origin in a sinking down of the womb below its normal position in the pelvis, and are unconnected with flexion of that organ, and that the lack of morbid symptoms in the second group of cases is explained by the fact that the uterus remains at its normal elevation.

1. *Traction upon the uterine supports.* The manner in which the womb is supported in the pelvis has been clearly demonstrated by Savage in his admirable investigations of the anatomy of the female pelvic organs.* He found by drawing down the womb with a vulsellum in the axis in which prolapse occurs, that tension was first exerted upon the utero-sacral ligaments. When, by the forcible stretching of these ligaments, the womb had descended about an inch in the pelvis, "some retaining agent, other than the broad ligaments, still prevented its arrival at this last stage (complete prolapse). The obstruction was found to be due to the sub-peritoneal pelvic cellular tissue, particularly where it surrounds and accompanies the uterine blood-vessels." It is only when this tissue is divided and the womb is drawn half through the vulva that the broad and round ligaments are put on the stretch. As long, therefore, as the womb remains at its normal

* The Surgery, Surgical Pathology and Surgical Anatomy of the Female Pelvic Organs. New York, 1880. Plates xviii. and xix.

elevation, it is supported principally by the utero-sacral ligaments and that portion of the pelvic cellular tissue which surrounds and accompanies the uterine blood-vessels.

The utero-sacral ligaments form a portion of the diaphragm of cellular tissue which, radiating horizontally in all directions from its uterine attachment, in a plane extending from the middle of the posterior surface of the pubic bone to the junction of the third and fourth sacral bones, forms the roof of the pelvis. The attachment of this diaphragm to the womb on all sides takes place at a point corresponding to the line of union of the cervical with the corporeal portion of the organ, or about opposite the internal os. Through this diaphragm the cervix projects downward into the vagina and the body upward into the abdominal cavity, each portion being virtually free from other attachment, since the broad and round ligaments are so lax that they are not put upon the stretch as long as the womb remains entirely within the pelvis. The womb is slung, as it were, upon a universal pivot, and therefore as long as the position of the point of support is unaltered, its axis may vary greatly in direction without producing any traction. But it is obvious that if, from any cause, the normal equilibrium between the uterine supports and the weight to be supported be destroyed, so that the womb sinks below its normal position, traction upon the utero-sacral ligaments and the pelvic cellular tissue will exist in exact proportion to the distance that the womb descends in the pelvis.

2. *Obstruction of the uterine circulation.* The womb receives its supply of blood through two arteries, the uterine and the ovarian. The uterine artery, a branch of the internal iliac, passes downward into the pelvis between the two layers of the broad ligament close to its iliac attachment. Upon reaching a point a little below the level of the os uteri, it curves inward and upward and comes into contact with the uterus just above the uterine insertion of the vagina. It then passes upward along the lateral border of the uterus, between the two layers of the broad ligament, giving off numerous horizontal branches which enter the womb at right angles. Upon reaching the superior angle of the uterus it anastamoses freely with the ovarian artery. The ovarian artery, the analogue of the spermatic artery in the male, is given off by the aorta just below the origin of the renal arteries. Passing downward to the pelvis it supplies the ovary. But numerous large branches ramify in the upper portion of the broad ligament and anastamose freely with the uterine artery at the upper angle of the uterus. Thus the womb is supplied with blood from above and below, the two sources of supply being supplementary to each other, as is proven by the fact that when either artery is abnormally small, as is not infrequently the case, the other is correspondingly enlarged. The venous arrangement is similar to the arterial. The blood passes from the uterus toward the heart by the uterine vein at about the level of the middle of the cervix and by the ovarian vein at the upper angle of the womb. These two veins are connected by a venous sinus, continuous with both, which passes upward along the lateral border of the uterus between the folds of the broad ligament, receiving at short intervals horizontal branches from the womb, and which is entirely devoid of valves. This sinus communicates with that of the opposite side by an intricate venous plexus within the uterine walls, extending without interruption from one side to the other, and from the os to the fundus.*

Since the uterine artery and the uterine vein follow the lateral border of the womb throughout its length and are closely bound to it by connective tissue, it is easy to see that any considerable flexion of the organ at any point will produce a sharp flexure in these vessels at that point and a diminution of their calibre. But it is difficult to understand how such a constriction can interfere in any degree with the circulation of blood in the womb, since both artery and vein give off lateral branches throughout their course, which render every horizontal segment of the womb independent of every other portion as respects its circulation. The circulation of the portion above such a constriction will still be carried on through the ovarian artery and vein, and that of the portion below the constriction through the uterine artery and vein, and each will be as perfect as though no flexion existed. At a late meeting of the London Obstetrical Society,

*Savage, opus cit., plates viii. and ix.

Dr. John Williams described the following experiment to test the influence of flexions in causing congestions. He stitched the fundus of the uterus closely to the cervix, thus securing the acutest flexion possible, and then injected a colored fluid into one of the veins of the broad ligament. Immediately the veins of the other side of the uterus became distended with the injection. On making a section, the whole of the veins in the uterus were found injected.* This proves what the arrangement of the vessels has already shown, that the acutest flexion does not interfere with the circulation of blood in the uterus.

A logical conclusion from these anatomical facts is that no obstruction can take place in the circulation of any part of the uterus from flexion of the organ, unless there be at the same time a constriction of the uterine or the ovarian blood-vessels at some point outside of the womb, that is, within the cellular tissue of the broad ligament. But if these two conditions co-exist, there will be a circulatory interference in the portion of the womb between the point of flexion and the point of constriction in the cellular tissue. No argument is needed to prove that traction upon an elastic tissue containing a blood-vessel will produce constriction of that vessel. We have seen that no such traction upon the cellular tissue exists as long as the womb remains at its normal elevation. But when it has descended to the distance of an inch, and not before, traction is exerted upon the pelvic cellular tissue, "particularly where it surrounds and accompanies the uterine blood-vessels." Hence in a case of flexion, accompanied by prolapse, we have the two factors necessary for interference with uterine circulation, while in flexion, unaccompanied by prolapse, one of the essential factors is absent.

3. *Traction upon the bladder*. It is commonly stated in the text-books that in flexions of the womb, pressure of the fundus upon the bladder produces irritation of that viscus. Of course this statement can apply only to anteflexion, since in retroflexion such pressure would be an anatomical impossibility. If pressure upon the bladder could produce vesical irritation, no woman would be free from this symptom. The normal condition of the womb

*American Journal of Obstetrics, September, 1885, p. 978.

is a state of anteflexion, so that the organ rests on the bladder. In addition to this, the bladder is always subjected to a varying amount of pressure from the weight of the superimposed intestines which rest upon it and follow it down as it contracts during micturition. Hence no bladder is ever free from pressure, and the extra weight of an anteflexed womb, which normally weighs only one and a half ounces, cannot produce any appreciable effect. The sensation which leads to a desire for micturition originates, according to Michael Foster,* in the circular muscular fibres within the bladder, especially the portion situated just posterior to the vesical termination of the urethra, sometimes spoken of as the vesical sphincter. As the bladder becomes distended by the accumulation of urine, such distention, when it has reached a certain point, is resisted by these circular muscular fibres. This muscular resistance, together with the escape perhaps of a few drops of urine into the urethra, gives rise to a desire to micturate, through a nervous mechanism which it is not necessary to describe here. Forcible mechanical expansion from any other cause will give rise to the same sensations and to spasmodic contractions of the bladder. If the bladder be empty, such spasmodic contractions will constitute vesical tenesmus, and if frequently repeated will soon become painful.

The relation of the uterus to the bladder is one of contact only, there being no cellular connection between the two viscera. Hence as long as the point of uterine support is undisturbed, flexion of the organ can produce no traction upon the bladder. But the vagina is closely connected to the bladder from its uterine insertion to the commencement of the urethra by a layer of cellular tissue, constituting with the walls of the two cavities the vesico-vaginal septum. Any descent of the womb is accompanied by a corresponding descent of this septum. Savage declares it to be a rule without exception that the bladder, notwithstanding the yielding character of its connection with the vagina, invariably follows the uterine cervix, whatever be the amount of downward displacement.† At the beginning of a uterine prolapse, it is obvi-

* Text-book of Physiology, London, 1878, p. 327.

† Opus cit., p. 83.

ous that the portion of the bladder dragged downward will be the point of its highest attachment to the vaginal wall, which is at the uterine insertion of the vagina. But as the prolapse increases, more and more of the bladder will become involved, until at length the traction will come to be exerted upon the portion constituting the internal sphincter. Since the anterior wall of the bladder is closely attached to the os pubis, traction upon the posterior wall will produce a forcible dilatation of the circular muscular fibres with its resulting sensation of a desire for micturition. As the degree of prolapse increases beyond this point, the amount of traction upon the bladder and of vesical disturbance will necessarily increase *pari passu.*

I have endeavored to demonstrate from an anatomical point of view that the symptoms accompanying those cases of uterine flexion which come under the notice of the physician are due to the sinking down of the womb in the pelvis, and not to the flexion. But such anatomical deductions are valueless unless confirmed by clinical observation. To complete the argument, it is necessary to show that when the womb is at its normal elevation in the pelvis such symptoms do not appear, and that when they are present the womb is found low down in the pelvis, and the symptoms are relieved by raising the organ to its normal elevation. A close and careful study of this subject, extending over several years of active practice, has convinced me of the truth of both these propositions. Since my attention has been directed to the subject, I have constantly been on the lookout for a case of flexion which presented the characteristic group of symptoms, but in which the womb had remained at its normal elevation. Thus far I have failed to find one such case. I have incidentally met with cases in which there was flexion with the womb high up in the pelvis. But in all such cases there were no subjective symptoms which would indicate its existence. Whenever I have found the group of symptoms which depend upon traction upon the uterine supports, obstruction of uterine circulation and interference with the functions of the bladder, I have always found the womb low down in the pelvis, and have also found that raising the organ to its normal elevation caused an abatement of such symptoms.

In all works upon gynæcology there is to be found a more or less incomplete recognition of these facts. But nowhere, except in a very recent article by E. C. Dudley,* do their full significance and their relation to the treatment of uterine flexions seem to be appreciated. It is interesting to note that the methods of treatment of flexions which have yielded the best results have been such as accord perfectly with this theory. Pessaries which raise the womb as a whole, such as those of Hodge, Hewitt and Gehrung, have been found more satisfactory than any others. It is also a significant fact that the methods prescribed for the treatment of anteflexion, retroflexion and prolapse are frequently quite identical. Mechanical conditions so diverse in themselves assuredly cannot be successfully treated by similar methods unless they possess some element in common. Clinical experience has thus led to the adoption of methods of treatment whose *rationale* has escaped recognition. Yet, although in a measure satisfactory in some cases, those methods have been so influenced by the erroneous theories upon which they are based that they have fallen far short of the degree of success that they might otherwise have attained. Consequently, they have never met with more than a half-hearted acceptance with the majority of the profession. Forty years ago, Meigs declared that "pessaries are necessary evils."† Twenty-five years later, Sims, with unconscious plagiarism, reiterated precisely the same words.‡ The practitioner of to-day endorses their dictum. The explanation of this seeming paradox is simple. The benefit derived from a pessary is exactly in proportion to its ability to raise the womb as a whole. The harm wrought by it is in proportion to the power expended in the endeavor to correct a flexion.

If the morbid symptoms usually attributed to flexion are due to prolapse of the womb, an important question relates to the causation of that prolapse. An uncomplicated case of flexion is rarely seen in practice. Not because such cases do not exist, but because they require no treatment. The uncomplicated cases

*Pepper's System of Medicine. Phila., 1886, Vol. IV., page 147, *et seq.*

† Females and their Diseases. Phila., 1848, p. 144.

‡ Clinical Notes on Uterine Surgery. New York, 1873, p. 264.

are seen only by accident; the complicated ones apply for relief. In every case of flexion, accompanied by marked symptoms, some complication will be found to exist. It may be a morbid growth in the womb, a stenosis of the cervical canal, a laceration of the cervix, a rupture of the perineum or a vaginal fistula. Any pathological condition which adds to the weight of the womb by increasing the amount of its solid tissues, or by inducing congestion, will cause it to settle down to a greater or less degree below its normal level. To consider all the causes which may produce this result would be to write a treatise on gynæcology, since nearly all morbid conditions of the pelvic viscera will give rise to some degree of increase of the weight of the womb. In nulliparous women the most frequent complication is stenosis of the cervical canal, which, by giving rise to obstructive dysmenorrhœa, induces a chronic congestion. In fertile women the most frequent complication is laceration of the cervix uteri, which increases the weight of the womb by interfering with the proper involution of the organ after labor.

It follows that in the treatment of uterine flexions there are two indications to be met: first, to raise the womb to its normal elevation; second, to remove the cause of increased weight. The elevation of the womb is to be regarded as the first step in the treatment, because there is present, as a rule, a greater or less degree of the so-called chronic pelvic cellulitis, whose removal is a necessary prerequisite to any safe and efficient operative procedure. Whatever view may be taken of the pathology of this condition, clinical experience has shown that it is not safe to divide or dilate an obstructed cervical canal, repair a lacerated cervix or even to introduce a sound to the fundus as long as it exists. The quickest and most efficient method for its removal, in common with the other symptoms of prolapse, is to raise the womb to its normal level in the pelvis and keep it there by appropriate mechanical support.

The normal position of the womb has been the subject of much discussion, and has never been satisfactorily determined, since scarcely any two authorities exactly agree upon this point. The womb is, within certain limits, a movable organ, since its position varies in different subjects and at different times in the same subject. It floats in the pelvis like a ship moored to a pier. As a ship so moored rises and falls with the tide and sways to and fro with the motion of the waves, so the womb rises and falls with the respiratory movements of the diaphragm and oscillates laterally and antero-posteriorly with the changes in the position of the body and the degree of distention of the bladder and rectum. Hence it is impossible to exactly define its normal position. But in every patient suffering with symptoms of prolapse, if the womb be not bound down by adhesions, it will be found that there is a certain point of elevation in the pelvis, to which if the womb be raised the symptoms are relieved. When this point has been found, the normal position of the womb has been determined for that individual case. To maintain this position is the proper function of mechanical support.

The question of how to support the womb by mechanical means is as old as the history of medicine. From the pomegranate, which Hippocrates inserted into the vagina for this purpose, down to the latest invention of the modern gynæcologist, the medical mind seems to have run riot in the multiplication of pessaries. Their almost infinite variety is the best evidence of their failure to accomplish the purpose for which they are intended. Thomas, after having invented thirteen different pessaries for the cure of anteflexion and anteversion, says: "Were I asked at the present moment whether I believed that in the aggregate they have accomplished more good or evil, I should be forced to give a doubtful reply."* The objections to pessaries in the treatment of flexions are manifold, and are universally recognized even by their most ardent advocates. It would therefore be superfluous to recount them here. The method which I recommended in my previous paper, and to which I have given the name of the "supporting tampon," is free from these objections. Its use is not debarred by the presence of so-called chronic pelvic cellulitis, nor by any amount of pain or tenderness in the roof of the pelvis. It is therefore applicable where pessaries would be entirely out of the question, and, even though applied by unskillful hands, is in-

*Diseases of Women, New York, 1880, p. 67.

capable of doing harm. It is indicated in any case in which the symptoms show the existence of traction upon the uterine supports, obstruction of uterine circulation or interference with the functions of the bladder. The relief which it gives to such symptoms is immediate, and places the patient in a very short time in a suitable condition for any operative procedure which may be necessary for the removal of the cause of the prolapse.

In order to properly apply the supporting tampon, it is necessary to first ascertain the position in the pelvis at which the womb is most free from pain. This may be done by raising the womb upon the index finger in the vagina while the patient stands. If the womb be bound down by adhesions this cannot be accomplished. But fortunately adhesions are rarely found. When the womb has been gently raised about as far as the finger can reach and held at that point for a short time, the patient will find that her pain and discomfort are greatly diminished. She must then be placed upon a table in Sims' position and Sims' speculum introduced. A pledget of cotton saturated with glycerine must be placed in the posterior *cul de sac*, another in the anterior fornix and a third and fourth on either side, so as to completely encircle the cervix. The size of these pledgets must be such as to fill the space between the cervix and the vaginal wall on all sides with a moderate degree of distention. The cervix will thus be held with a tolerable degree of firmness in its normal axis. A larger pledget of cotton, also wet with glycerine, must then be placed immediately below the cervix, so that it will lie between it and the vaginal promontory. The size of this pledget must be such that when in position it will raise the womb to the point where it has been found to be most free from pain and, resting upon the vaginal promontory, will form a platform of support for the organ. The patient may then resume the standing position, and if the tampon be properly applied she will be sensible of immediate relief from its application. A certain amount of experience is necessary in order to insert the tampon in such a manner that it will remain where it is placed and hold the womb in the desired position. If it be packed too firmly around the cervix, the natural mobility of the womb will be abolished and the jar of every motion will be felt in the pelvis by the patient. On the other hand, if too loosely applied, it will be found in a few hours to have fallen down into the lower part of the vagina and all its usefulness will be gone. Experience alone will enable the operator to avoid these two extremes.

In my former article upon this subject, I stated that "a tampon of cotton should never be allowed to remain in the vagina for more than twenty-four hours" on account of its interference with the daily use of the vaginal douche of hot water which I considered an indispensable adjunct to the treatment. Further experience has convinced me that I overrated the value of the daily douche, and that patients improve with equal rapidity under a less frequent use of it. I was led to experiment in this direction on account of the objection made by patients to the trouble and expense incident to daily treatment. I have found that if properly applied the tampon will, as a rule, retain its position for forty-eight hours, and the disinfectant properties of the glycerine will prevent its becoming foul in that time. It should then be removed, a hot vaginal injection used and a new tampon inserted. Under this treatment all tenderness and engorgement in the cellular tissue will rapidly subside, the womb will return to its normal weight and size, endo-cervicitis and leucorrhœa will cease, menstruation will become normal, pain in the back, loins and pelvis will disappear. The rapidity with which these changes will take place will be of the nature of a revelation to one who uses the supporting tampon for the first time.

The second indication in the treatment, the removal of the cause of increased weight of the womb, it would be superfluous to notice at length, since it involves processes whose description is found in every text-book of gynæcology. Suffice it to say that whatever may be the complicating pathological condition whose presence is the cause of increased weight of the uterus, it must be remedied by appropriate therapeutic or operative measures. The beneficial results obtained by the use of the supporting tampon will thus be rendered permanent. If the use of the tampon be discontinued without fulfilling this second indication, it will be only a question of time when the original condition will return, and the patient will be as badly off as at the beginning of the treatment. In other words, the supporting tampon is a palliative and preparatory measure merely. To expect more of it would show a lack of appreciation of the principles involved in its use.

